# Medical Opinion and Movement

**Published:** 1910-07-30

**Authors:** 


### Medical Opinion and Movement.

### Dilatation for Prostatic Hypertrophy.

A case in which Bayer's method of dilatation of the prostate for hypertrophy of the organ was successfully carried out is reported by Kraemer in the *Deutsche Med. Woch.* The patient, who for five days had been troubled with partial retention, was suddenly seized with complete retention. Progressive dilatation with bougies of the prostatic urethra up to a No. 44 of the French scale sufficed to re-establish the normal flow. According to Bayer all cases of abscess with an elevated temperature and purulent cystitis must be excluded from this form of treatment, but nevertheless, as in the present case, there is no contra-indication to its use when an intense cystitis is capable of improvement. It would seem from the simplicity of the operation that it should be tried in all suitable cases before more radical procedures are commenced. After all due aseptic precautions have been taken, the method may give good results, and should at all events be tried thoroughly before a negative opinion as to its merits is given.

### Typhoid Fever in Infants.

Rommeler, in the *Münch. Med. Wochen.*, reports a case in which a seemingly healthy infant transmitted typhoid fever to a third person. The child's mother had been removed to hospital suffering from typhoid fever, the child being sent to a friend to be looked after. With the exception of a slight attack of diarrhœa, which soon passed, the infant seemed to be in perfect health. Two weeks later, however, the foster mother herself developed typhoid fever, and in turn her three children, her niece, three years old, and a servant were attacked. On examining the blood of the infant a positive Widal's reaction was obtained, and on one occasion active typhoid bacilli were isolated from the stools. The author points out that when a nursing mother develops typhoid fever her infant should also be isolated, for symptoms of the disease are often so slight in young children that there is a danger of an apparently healthy baby carrying infection to others.

### Effect on Eyes of Various Rays.

For some time past a number of ophthalmologists have accused the ultra-violet rays of causing injury to the eyes, and as the intensive methods of illumination adopted nowadays produce these rays in considerable quantity they advise the use of special glasses to protect the sight from these hurtful radiations. Professor Best, of Dresden, in the *Med. Klin.*, in the course of a long and minute study of this question, concludes that if it be true that ultra-violet rays, like luminous rays of certain intensity, are capable of injuring the eyesight, nevertheless in practice it is above all the hurtful action of the luminous rays which predominates, and that, in consequence, it is necessary before everything else to protect oneself from an excess of intensity of the rays, by means of spectacles, the object of which is to lessen the force of the radiations, and which, therefore, should be of a grey colour. His advice is not, however, the same when dealing with patients suffering from eye diseases, especially those of the middle of the eye or retina. Then there is no longer question of an objective excess of luminosity, but of an excess of retinal sensibility. Since in these cases the retina is particularly sensitive to the rays of the spectrum, which are richest in yellow, patients should be advised the use of blue glasses.

### Alum and Ingrowing Nails.

According to the *American Journal of Clinical Medicine*, every case of ingrowing toe-nail can be cured in five days by the free application of dry powdered alum. No pain attends this form of treatment, and the destruction of the diseased tissue results in the formation of a hard resistant non-sensitive bed for the nail with a cure of the ingrowing tendency. The non-toxicity of the alum, its easy application, and the good results obtained from it render it the treatment of choice, at least in cases where no operative measures are contemplated. A soap-and-water fomentation is first applied for twenty-four hours, and then the alum is applied to the space between the nail and its bed; a tampon of cotton-wool is next placed on the alum, and the applications repeated daily. Suppuration rapidly ceases, the parts dry up, and pain and discomfort vanish almost at once. At any rate, the method would seem worthy of trial.

### Huge Ovarian Cyst in Hindu Woman.

A remarkable case of ovarian cyst occurring in a Hindu woman, aged twenty, is reported by Rao Saheb Dr. P. R. Bhandarkar in the *Indian Medical Journal.* Six years previous to admission to hospital the woman had had an attack of fever, after which she noticed a swelling in the neighbourhood of the umbilicus, which gradually grew. A year later menstruation ceased and had never since been resumed. On admission, the abdomen was found to be much enlarged, but the swelling was not symmetrical. Two marks of cauterisation were present near the umbilicus, and the superficial veins of the abdomen were distended. In the dorsal position a hard mass could be felt at the epigastrium, and percussion yielded a dull note all over the abdomen, except in the flanks. Some idea of the size of the tumour can be obtained from the following measurements. The distance between the umbilicus and ensiform cartilage was 14 inches, while 20 inches separated the umbilicus from the right, and 19 inches the same point from the left, anterior superior iliac spine. The circumference of the abdomen at a point midway between the umbilicus and the symphysis pubis was 55 inches. Lieut.-Col. J. R. Roberts, I.M.S., opened the abdomen under an anæsthetic by a vertical incision three inches in length midway between the umbilicus and symphysis pubis. The fluid from the cyst was evacuated by a trocar and cannula. The cyst wall was found adherent to the mesentery, parietal peritoneum, and omentum, and needed careful dissection to free it from these. The amount of fluid removed measured 95 pints, and weighed 120 pounds, the weight of the cyst wall being 10 pounds 2 ounces. After operation the patient remained weak for a few days, but in five weeks she was sufficiently recovered to be discharged. The loose skin of the abdomen rapidly contracted, and when she left hospital was but little too large for the reduced size of her abdominal contents.

### Idiopathic Osteo-myelitis of Upper Jaw.

Zamenhof, in the *Medycyna i Kronika Lekarska*, reports a case of idiopathic acute osteo-myelitis of the superior maxilla in a child three years of age with a negative family history. A swelling of the cheek, accompanied by high fever, had appeared two months before the child was seen by the author, but in a few days this had quieted down, a persistent pain in the upper jaw remaining however. Later pus collected and small fragments of bone became detached from the superior maxilla. At the examination a swelling of the cheek was found with complete necrosis of the right upper alveolar process. No communication with the antrum of Highmore was found. After removing the sequestrum with forceps the child recovered in a week. The author considers that the case must be one of idiopathic osteo-myelitis, as no history could be obtained of any antecedent infectious disease, or of the presence of the disease at any other time, while the progress of the case would seem definitely to exclude syphilis as a cause of the necrosis. He was only able to find three analogous cases in the literature.

### Chronic Intussusception of Adults.

Of this condition as a primary lesion independent of previous ulceration, tumours, and tuberculosis, Dr. Goodall has collected 122 cases, and has analysed them for the *Boston Medical and Surgical Journal*. The condition is less rare, according to this author, than is commonly supposed, for it commonly escapes diagnosis, and many of the reported cases were discovered only at autopsy. The clinical picture, however, is fairly suggestive as a rule, and the mortality under proper surgical treatment should be low. Pain is the most frequent symptom, often as a sudden colic which disappears as rapidly as it comes on. Such cessation may be due to disinvagination in the early stages of the complaint. Later on pain is seldom entirely absent between the attacks, which occur at short intervals, from a few hours to two or three days. The pain is not as a rule definitely localised, and may be evoked by food, defæcation, or exercise. Vomiting is seen in about half the cases; there is nothing especially characteristic about it. Constipation and diarrhœa are about equally common, and the stools in the latter case are sometimes almost dysenteric; blood is not at all unusual, but pus is seldom seen. There is a continuous loss of weight in most patients. The abdomen is as a rule flat or retracted, not distended; peristalsis may be visible, as also distended coils of intestine in localised areas. An abdominal tumour can be detected in nearly every case, though not at every examination, and sometimes only under an anæsthetic. The mass may change in shape and position from day to day, and may disappear as the pain is relieved; this is an important point in diagnosis.

### Formalin for Ringworm.

Since the introduction of the $x$-ray treatment of ringworm the various antiseptic and parasiticide remedies have fallen somewhat into the background. Dr. Mackinnon, who writes from Cape Colony, publishes in the *Lancet* a brief description of a method in which he has great faith; it seems well worth trial by those who, as he presumably is, are not within reach of a thoroughly equipped electrical plant. The drug he recommends is not mentioned in the latest edition of the standard British work on skin diseases, and practitioners in Great Britain may therefore not be familiar with it. The area of skin affected is washed with spirit and then allowed to dry. Then with a soft camel-hair brush a 40-per-cent. solution of ordinary formalin is painted on, and the head is meanwhile placed in such a position that the liquid will not run off. According to the author, one such application is sufficient as a rule to effect a cure, but if there is any doubt he repeats the application in five days. There is sometimes some smarting in young children, and the skin often scales off from the irritating action of the drug; but these are only slight drawbacks from a process which is described as clean, rapid, and effective.

### Abdominal Auscultation.

Dr. V. Piazza-Martini, of Palermo, advocates auscultation of the abdomen as a means to determine the presence of adhesions in cases of large abdominal tumours. In regard to their removal by operation, it is of considerable prognostic importance to decide whether such tumours are free or adherent; but, as the author points out, the ordinary signs, such as transverse mobility, are often indefinite and inconclusive. According to Dr. Piazza-Martini, however, auscultation of the abdominal wall over the tumour gives a definite sign. If the mass is adherent the heart-sounds are heard clearly over the adherent area. Often, too, the respiratory murmur and vocal resonance may also be transmitted. In three cases of ovarian cyst the signs obtained by auscultation were confirmed by operation. In all these cases the cardiac sounds were clearly transmitted over a large area of the abdominal wall, and in two cases the respiratory murmur and vocal resonance were heard also, though less distinctly. On operation the cysts were found to be extensively adherent over the areas where the sounds were transmitted. In two other cases, again, in which these signs were not obtainable by abdominal auscultation, no adhesions were found on operation, the tumours being quite free.

Another sign obtained by abdominal auscultation has been discovered by Dr. Wilms, and may prove of considerable diagnostic value in doubtful cases of intestinal obstruction. An account of this sign is given in the *Münch. Medizinische Wochenschrift.* When the intestine is obstructed at a certain point, fluid and gas tend to accumulate above the point of obstruction. The intestine is distended by the contents, and the level of the fluid being constantly changed with the peristaltic movements, bubbles of gas break on the surface of the fluid. Owing to the tension of the intestine this breaking of bubbles of gas gives rise to " bruits " of a metallic nature, and these metallic bruits can be heard clearly on auscultation of the abdominal wall. According to Dr. Wilms this sign can be obtained at quite an early period of obstruction, when the intestine is not sufficiently distended to produce the better known borborygmi sounds. The thickness of the abdominal wall does not prevent these bruits from being transmitted, so that they can be heard quite well even in obese subjects. The author finds that these metallic bruits are a valuable diagnostic sign in cases of intestinal obstruction, as not only is their presence conclusive evidence of the condition, but ileus may be excluded on their absence. He gives details of several cases in which other signs were doubtful, and the diagnosis of obstruction made on account of the presence of the metallic bruits was confirmed by operation. On the other hand, in the case of a young man suffering from constipation and vomiting for a week, the absence of metallic bruits enabled him to diagnose a condition of enterospasm, which completely cleared up with ordinary medical treatment. In all such abdominal conditions an early diagnosis is of paramount importance, and, according to the author, this sign may be obtained at a time when meteorism, vomiting, and borborygmi with colic have not yet developed; it should therefore prove of considerable service to the clinician.

### Anti-Typhoid Vaccine.

At a meeting of the Académie de Médecine Dr. H. Vincent gave an account of the anti-typhoid vaccine he has succeeded in preparing and of its effects in the human subject. He employs as antigen the " autolysates " of living bacilli in physiological saline solution, clarified by centrifugalisation and sterilised by agitation with ether. The ether is got rid of by evaporation at 37° C. for a few minutes. This vaccine is found to be highly protective for animals. For man he has prepared a polyvalent vaccine from eight strains of typhoid and paratyphoid bacilli. It has been injected in thirteen adults under the skin of the flank. It is well tolerated, and only produces slight local pain without fever. Four injections are made at intervals of a week or ten days. This vaccine contains, according to the author, the endotoxins and exotoxins of the typhoid and paratyphoid bacilli—that is to say, the essential and active biological principles of these microbes—and excites the formation of an abundant quantity of defensive anti-bodies in the subjects vaccinated. The agglutinating power amounts to between $\frac{1}{60}$ and $\frac{1}{1000}$. The bacteriolytic power is very intense. Placed in contact with the serum of a vaccinated subject, the typhoid bacillus becomes granular, deformed, irregular, and almost unrecognisable. The bactericidal power of the serum of a vaccinated subject is almost complete at 1 in 1,000, and is quite appreciable at 1 in 2,000, and even sometimes at 1 in 5,000. It does not produce a negative phase. As it is neither heated nor contaminated with antiseptic it preserves its efficacy.

### Bone Transplantation.

An interesting case of bone transplantation is recorded by Dr. Rovsing in the *Hospitalstidende*, in which the upper third of the humerus was replaced by a portion of the fibula of the patient. The patient, aged twenty-six, was affected with an osteosarcoma of the upper third of the humerus. By a semi-circular incision around the acromion process this shoulder-joint was opened. From the centre of the incision a second incision was carried down the external aspect to the middle of the arm. The upper end of the humerus and shaft were then exposed and isolated, but the periosteum and muscular insertions were left adherent. The bone was then resected at the junction of the middle and lower thirds. The wound was plugged and the upper part of the right fibula rapidly exposed and resected, so that the portion removed, including the head, was 3 centimetres longer than the resected piece of humerus. Muscular insertions were left attached to the bone. The end of the fibula was pointed so as to fit into the medullary canal of the humerus. The head of the fibula was placed into the glenoid cavity and the capsule sutured. The muscles were also sutured to the muscular attachments on the fibula. The wound healed well. Two months after operation radiography shows that the fibula has remained in position and has not atrophied. The movements at the shoulder-joint are quite free, and the patient can move the arm sufficiently for feeding, brushing the hair, etc., only there is some limitation in the movement of abduction. The patient has normal use of the leg and suffers no inconvenience from removal of the fibula.

### Bacteriology of Normal Lochia.

The lochia of twenty perfectly normal puerperal patients have, according to *Folia Ginecologia*, been recently examined by Cova with a view to ascertaining the bacteriological condition of the secretions. He found that in the physiological uterus no germs can be obtained from the uterine cavity though they are constantly present in the vagina and vestibule. For the first twenty-four hours the lochia are quite sterile; later germs appear, but they are only those commonly found in the vagina. Only some of the germs found in the vestibule are to be obtained from the vagina—*e.g.* the bacillus coli is rarely met with in the latter organ, while common at the vulva. The vaginal flora consist of various kinds of cocci, especially diplococci, non-virulent and saprophytic. The vestibular flora are more numerous, including as they do organisms from within, and from without such as the bacillus coli and the skin-organisms.

